# Psychometric evaluation of a Swedish version of Minneapolis-Manchester quality of life-youth form and adolescent form

**DOI:** 10.1186/1477-7525-11-79

**Published:** 2013-05-08

**Authors:** Eva-Lena Einberg, Ibadete Kadrija, David Brunt, Jens N Nygren, Petra Svedberg

**Affiliations:** 1 School of Social and Health Sciences, Halmstad University, Halmstad SE - 301 18, Sweden; 2 School of Health and Caring Sciences, Linnaeus University, Växjö, Sweden

**Keywords:** Children, Cancer, Questionnaire, Reliability, Validity

## Abstract

**Background:**

It has become important to measure long-term effects and quality of life in survivors of childhood cancer. The Minneapolis- Manchester Quality of Life (MMQL) instrument has been proven to better capture the quality of life (QoL) perspective of health than other instruments. The instrument has age appropriate versions and is therefore favourable for longitudinal studies of QoL of children surviving from cancer. The aim of this study was to evaluate the psychometric properties of the Swedish version of MMQL-Youth Form and the Adolescent Form focusing on: 1) face and content validity 2) the internal consistency and 3) the test-retest reliability.

**Methods:**

The sample consisted of 950 pupils (11–16 years old) from 7 schools in the western Sweden who completed the questionnaire. For the test-retest evaluation 230 respondents completed the questionnaire two weeks later.

**Results:**

Face and content validity was supported and internal consistency was found to be acceptable for the total scale for both the MMQL-Youth Form (8–12 years of age) and the Adolescent Form (13–20 years of age). Test-retest reliability for the MMQL-Youth Form was moderate for 50% of the items and good for the remaining. For the MMQL-Adolescent Form the test-retest showed moderate or good agreement for 80% of the items and fair for 20%.

**Conclusions:**

The result indicated that the Swedish version of the MMQLYouth Form and Adolescent Form was valid and reliable in a sample of healthy children in a Swedish context. It is recommended to test the instrument among diverse samples of children such as survivors of childhood cancer in order to validate its usefulness in research and clinical settings.

## Background

Improved treatment programs and care over the past thirty years have substantially increased the proportion of children surviving cancer and today approximately 80% survive long term [1]. However, surviving cancer during childhood imposes a number of physical and psychosocial difficulties later in life. These include school and work-related problems, difficulties related to friendships and intimate relationships, sleep disturbance, infertility and physical and emotional distress. These problems can have an immediate and negative impact on the quality of life (QoL) later in life [2]. It has thus become important to measure long-term effects and quality of life of survivors of childhood cancer [1].

The concept of QoL is mostly based on literature concerning adults [3]. The World Health Organization defines QoL as “*the individuals’ perceptions of their position in life in the context of the culture and value systems in which they live and in relation to their goals, expectations, standards and concerns”*[4]. QoL is a broad concept of general well-being, broader than health related quality of life (HRQoL) that is more specific and refers to the impact of health and illness [2,3,5]. However, the perceptions of children and young people of their health and QoL differ from those of adults. Important factors in relation to QoL for young people are developmental stage and relationships with friends and family [3]. It is also well known that children and parents rate the child’s health and QoL differently. Measuring children’s QoL by asking parents or another proxy respondent can only supplement but not replace responses from the child him/herself [6-8]. Furthermore, the concept of QoL reflects the views of the individual (WHO, 1998) and thus the individual, in this case a child or a young person, should as far as possible rate his/her own QoL.

Various generic or disease-specific instruments exist for measuring QoL or HRQoL among children and adolescents [9,10] and in recent years there has been an increase in the number of instruments, particularly disease-specific instruments [10]. Examples of generic instruments are the Child Health Questionnaire (CHQ-87), the PedsQL 4.0 and the KIDSCREEN-52 questionnaire [9,11]. Examples of instruments developed as disease-specific are the Pediatric Oncology Quality of Life scale (POQOL), PedsQL 3.0 cancer module and the Minneapolis-Manchester Quality of Life instruments (MMQL) [9], however these instruments have also proved to capture generic aspects of QoL and HRQoL [9]. Generic instruments allow comparison between groups, such as cancer survivors and the general population, and across different conditions or settings. If the objective is to measure the impact of illness on a person’s life, generic instruments lack the sensitivity and may not adequately cover specific concerns for, for example, cancer [2,10].

When assessing the HRQoL of children, their perception of health and QoL, their developmental changes and their cognitive function, are factors that should be considered [3,6]. Savage et al. [6] found three disease-specific measures (MMQL-Youth Form, PedsQL™4.0, Pediatric Cancer Quality of Life-32) with sound psychometric properties in their review of QoL in children with acute lymphoblastic leukemia and only MMQL-Youth Form and PedsQL included age appropriate versions [6]. The MMQL instrument has, in comparison with other health status and QoL instruments developed for children, proved to better capture the QoL perspective of health [9]. Furthermore, the items used have a mainly positive phrasing, which is important for how each item is perceived and for the child’s experience of contributing to the questionnaire [9].

The MMQL instrument was developed in three versions to meet developmental needs of different age groups and is based on extensive exploratory work with children [12,13]. The MMQL-Youth Form was created for evaluation of children (8–12 years of age) [13] and the MMQL-Adolescent Form for evaluation of adolescents (13–20 years of age) [12] surviving from cancer. There is also a version for adult individuals between 21–45 years of age [12,14]. The questionnaire consists of 4 subscales in the Youth Form [13], and of 7 subscales in the Adolescent Form [12]. Internal consistency reliability has been reported for the MMQL-Youth Form (overall alpha = 0.85, range 0.72 - 0.80) [13], and for the MMQL-Adolescent Form (overall alpha = 0.92, range 0.67 - 0.89) [12]. The test-retest reliability has been reported for MMQL-Youth Form as ranging from 0.56 to 0.79 and for the total scale as 0.72 [13], and for the MMQL-Adolescent Form ranging from 0.60 to 0.90 and for the total scale as 0.71 [12].

The MMQL-instrument has been used in surveys in the United States [14,15], the UK [16] and in Denmark [17]. In the UK they performed an adaptation of the MMQL instrument with healthy children, children with chronic conditions and children with cancer between 8–18 years of age [16] and the Danish version of the MMQL-instrument was used in a survey with survivors of childhood brain tumors [17]. Wu et al. [15] compared adolescents with cancer, off-therapy survivors and patients on therapy, with healthy controls. They selected the MMQL instrument because they found the items generic enough to be administered in its entirety on healthy controls [15]. Thus, as the MMQL instrument has been applied to numerous conditions involving both healthy children and children affected by or with experience from disease [6,15,16] it can be used for evaluation of generic aspects of health. According to Streiner and Norman [18] a questionnaire must be re-evaluated when it is used on a different sample or when it is translated into another language. The aim of this study was thus to evaluate the psychometric properties of the Swedish version of MMQL-Youth Form and the Adolescent Form on school children focusing on: 1) face and content validity 2) the internal consistency and 3) the test-retest reliability.

## Methods

### Design and settings

The present study had a methodological design where the translated version of the MMQL-Youth Form and Adolescent Form was psychometrically tested. The study design was approved by the local ethics board at Halmstad University (Dnr 90-2011-2863) and the principals at the participating schools. Data collection was carried out during autumn 2011 in a sample of primary schools in western Sweden.

### Questionnaire

The MMQL-Youth Form is a 32-item self-rating questionnaire and consists of four quality of life domains (subscales); physical symptoms, physical functioning, psychological functioning and outlook on life/family dynamics [13]. The MMQL-Adolescent Form is a 45-item self-rating questionnaire and consists of seven quality on life domains (subscales); physical functioning, cognitive functioning, psychological functioning, body image, social functioning, intimate relations and outlook on life [12]. The items in both the MMQL-Youth Form and the Adolescent Form have a 4 or 5 point Likert scale and higher scores indicate greater HRQoL [12,13].

### Translation procedure

The original versions of MMQL-Youth Form and Adolescent Form were translated into Swedish, according to forward-backward methodology [19,20]. Two researchers, both native Swedish speakers and fluent in English, translated the questionnaire to Swedish and then a third researcher, a native English speaker, also fluent in both languages, with no previous knowledge of the original questionnaire, retranslated it into English. The small differences between the original version and the retranslated version, which were of a cultural and linguistic nature, were then discussed in the research group in order to improve the quality of the Swedish translation and to reach consensus. The items in the Swedish version were then discussed in the research group as well as with 20 children between 8–15 years of age, both girls and boys. The phrasing of some of the items was further adjusted based on feedback from the children who had found these to be vague or confusing.

### Recruitment and data collection

Children from two age groups, 6^th^ year of primary school and 9^th^ year of primary school, were recruited from seven schools in a municipality of 92 000 inhabitants. In the municipality approximately 14% of the population was foreign-born, the unemployment rate was 7%, while 9% of the inhabitants received sickness benefits or activity compensation and about 1300 individuals received welfare benefits (Statistics Sweden, 2011). There are 42 schools in the municipality. The schools included in the study were located in central and suburban areas of the municipality, had children in both 6^th^ and 9^th^ year age groups and more than 100 children in total. Seven schools met the inclusion criteria and were selected for inclusion in the study. The included schools also represent different socio-demographic area in the municipality; low-and high-income earners, a variety of housing and domestic-and foreign-born inhabitants. A total of 24 classes in 6^th^ year (n = 536 children) and 25 classes in 9^th^ year (n = 576 children) were included. The principals at each school were contacted for approval for the participation and parents were then informed about the purpose and structure of the study. Participation was voluntary and if children or their parents declined to participate, they could decide not to fill in the questionnaire without having to explain why. The questionnaires, including a return envelope, were distributed to the respondents after information was given in the class room. Children who declined to participate returned the questionnaire without providing details. The questionnaire was completed directly and collected by the researcher, except for two schools where the teachers distributed and collected the questionnaires in return envelopes.

The sample consisted of 950 respondents (469 in 6^th^ year and 481 in 9^th^ year), who agreed to participate and completed the questionnaires (response rate 88% and 84% respectively). For demographic characteristics of the sample see Table 1. For test-retest evaluation two weeks later, questionnaires were administrated by teachers to the children in 13 of the 49 classes. A total of 127 children in 6^th^ year and 163 children in 9^th^ year were asked to complete the questionnaire a second time (response rate 87% and 74% respectively).

**Table 1:** Demographic characteristics of the study population

	**Grade 6**	**Grade 9**
	**n**	**n**
**Age**	469	478 (missing n = 3)
11 or younger	9 (1.9%)	
12	440 (93.4%)	
13	16 (3.4%)	
14	4 (.9%)	84 (17.5%)
15		375 (78%)
16		19 (4%)
**Gender**	467 (missing n = 2)	478 (missing n = 3)
Female	232 (49.5%)	224 (46.6%)
Male	235 (50.1%)	254 (52.8%)
**Country of birth**	468 (missing n = 1)	479 (missing n = 2)
Sweden	409 (87.2%)	421 (87.5%)
Foreign born	56 (11.9%)	57 (11.9%)
Don’t know	3 (.6%)	1 (.2%)
**Parents’s country of birth**	455 (missing n = 14)	472 (missing n = 9)
Both parents in Sweden	282 (60.1%)	298 (62%)
One parent born abroad	59 (12.6%)	60 (12.5%)
Both parents born abroad	114 (24.3%)	114 (23.7%)
**Siblings**	469	478 (missing n = 3)
0	23 (4.9%)	30 (6.2%)
1	192 (40.8%)	202 (42%)
2-3	202 (43.1%)	199 (41.3%)
4 or more	72 (11.1%)	47 (9.7%)

### Statistical analysis

The MMQL-Youth Form and Adolescent Form were next examined for face and content validity, internal consistency and test-retest reliability. All the respondents who completed the questionnaire were also asked to evaluate the questions for clarity and readability in order to ascertain face and content validity [21]. *Cronbach’s alpha coefficient* was used to calculate the internal consistency of the two questionnaires and the subscales and was deemed acceptable if alpha ≥ .70 was achieved [22]. *Intraclass Correlation Coefficients (ICC)* were calculated for each item in the instrument in order to investigate test-retest reliability. The ICC produces a value of 1.0 only when the scores on the first occasion are exactly the same as those on the second occasion. The reference values for the levels of agreement consider < 0.20 as poor agreement, between 0.21-0.40 as fair, 0.41-0.60 as moderate, 0.61-0.80 as good and between 0.81-1.00 as very good agreement [18]. Statistical analyses were performed using the SPSS software 20.0 (SPSS Inc. Chicago, IL, USA).

## Results

The results concerning the face and content validity showed that the respondents understood the statements in the questionnaire and assessed that they had sufficient clarity and readability. Furthermore they evaluated the items as being relevant for the focus of the measure, for example one respondent wrote “It’s good that the questions are about how one feels about one’s health”.

The Cronbach*’*s alpha coefficient for the total scale was 0.88 for the MMQL-Youth Form (subscales from 0.66 to 0.81) and 0.92 for the MMQL-Adolescent Form (subscales from 0.69 to .91) (Table 2).

**Table 2:** Cronbach’s alpha coefficient for the MMQL-Youth form and adolescent form

***Sub-scales of the scale***	***Youth form***	***Adolescent form***
	***Cronbach’s alpha coefficient***	***Cronbach’s alpha coefficient***
Physical functioning	.66	
Psychological functioning	.81	
Physical Symptoms	.68	
Outlook on life/family dynamics	.78	
Physical functioning		.69
Cognitive functioning		.86
Psychological functioning		.83
Body image		.78
Social functioning		.85
Outlook on life		.91
Intimate relations		.75
***MMQL overall scale***	.88	.92

The test-retest reliability according to ICC ranged from 0.43 - 0.78 for the original instrument MMQL – Youth Form (Table 3). Sixteen of the items (50%) showed good agreement and the remaining 16 items showed moderate agreement between the two occasions.

**Table 3:** Intraclass correlation coefficients (ICC) for the original instrument MMQL – Youth Form (n = 110)

***Item***	***n***	***ICC***	***95% CI***
1. I do as well as my friends in sports	108	0.527	0.38 - 0.65
2. I have a lot of energy	107	0.654	0.53 - 0.75
3. I have a lot of energy for running or sports	105	0.759	0.67 - 0.83
4. I cannot do many activities because of my health	107	0.552	0.41 - 0.67
5. I cannot do many activities because of problems with my arms or legs	107	0.490	0.33 - 0.62
6. In games and sports, I like to watch rather than take part	107	0.567	0.42 - 0.68
7. Sad?	108	0.527	0.38 - 0.65
8. Angry?	108	0.637	0.51 - 0.74
9. Lonely?	108	0.464	0.30 - 0.60
10. Frightened?	107	0.648	0.52 - 0.75
11. Worried about dying?	107	0.710	0.60 - 0.79
12. Worried about your health?	108	0.471	0.31 - 0.61
13. Worried about things in general?	104	0.524	0.37 - 0.65
14. Not as good as most people?	107	0.432	0.27 - 0.57
15. My parents treat me in the same way they treat my brothers and sisters	105	0.571	0.43 - 0.69
16. My parents are usually patient with me	103	0.759	0.66 - 0.83
17. feel different from your friends	106	0.508	0.35 - 0.64
18. Do you have pain or discomfort in your stomach or tummy?	106	0.515	0.36 - 0.64
19.Do you have headahes?	107	0.637	0.51 - 0.74
20. Do your arms and legs ache?	108	0.634	0.51 - 0.73
21. Do you have discomfort in yor chest during active exercise?	106	0.600	0.46 - 0.71
22. Do you get pains that wake you up at night?	107	0.660	0.54 - 0.76
23. Do you have difficulty with your hearing?	108	0.713	0.61 - 0.78
24. Do you have difficulty with your talking (e.g., stuttering/stammer)?	107	0.731	0.63 - 0.81
25. Do you have difficulty seeing clearly (even wearing glasses)?	107	0.481	0.32 - 0.61
26. Do you have difficulty falling asleep?	108	0.686	0.57 - 0.78
27. I am looking forward to the future	108	0.630	0.50 - 0.73
28. I am happy the way things are	109	0.557	0.41 - 0.67
29. I am happy with the state of my health	108	0.648	0.52 - 0.75
30. I am happy with my life in general	109	0.781	0.70 - 0.85
31. I am a healthy person	109	0.775	0.69 - 0.84
32. I expect to live a long life. I expect to grow old	108	0.584	0.44 - 0.70

The ICC for the MMQL – Adolescent Form ranged from 0.24 - 0.76 (Table 4). Ten of the items (22%) showed good agreement, 26 items (58%) showed moderate agreement and 9 items (20%) showed fair agreement between the two occasions.

**Table 4:** Intraclass correlation coefficients (ICC) for the original instrument MMQL – adolescent form (n = 120)

***Item***	***n***	***ICC***	***95% CI***
1. I have a lot of energy	119	0.477	0.33 - 0.61
2. I need time out to rest during the day	118	0.532	0.39 - 0.65
3. I have a lot of energy for running or sports	118	0.611	0.49 - 0.71
4. I cannot do many activities because of my arms or legs	118	0.346	0.18 - 0.50
5. I cannot do many activities because of my health	119	0.505	0.36 - 0.63
6. In games and sports, I like to watch rather than take part	119	0.529	0.39 - 0.65
7. Sad	119	0.698	0.59 - 0.78
8. Angry	119	0.587	0.46 - 0.69
9. Tired during the day	119	0.571	0.44 - 0.68
10. Lonely	118	0.686	0.58 - 0.77
11. Frightened	119	0.429	0.27 - 0.57
12. Anxious or nervous	117	0.433	0.27 - 0.57
13. Strong and healthy	117	0.370	0.20 - 0.52
14. Worried about dying	119	0.637	0.52 - 0.73
15. Worried about my health	118	0.396	0.23 - 0.54
16. Worried about things in general	115	0.331	0.16 - 0.48
17. Not as good as most people (inferior to them)	119	0.361	0.20 - 0.51
18. How satisfied are you with your weight?	118	0.447	0.29 - 0.58
19. How happy are you with the way you look?	115	0.571	0.43 - 0.68
20. How do you feel about your body development right now?	114	0.248	0.68 - 0.41
21. I like my body the way it is	118	0.590	0.46 - 0.70
22. When others look at me they think that I am poorly developed	116	0.620	0.49 - 0.72
23. I am uncomfortable with the way my body is developing	118	0.385	0.22 - 0.53
24. I find it difficult to make friends	118	0.529	0.39 - 0.65
25. I feel left out in groups of people my own age	117	0.549	0.41 - 0.67
26. People like to be with me	116	0.538	0.39 - 0.66
27. I have a lot in common with my friends	117	0.475	0.32 - 0.60
28. I get along well with people own age	118	0.374	0.21 - 0.52
29. I have many close friends	117	0.581	0.45 - 0.70
30. I have similar hobbies and interests to those of people my own age	118	0.648	0.53 - 0.74
31. Being togheter with other people gives me a good feeling	118	0.357	0.19 - 0.51
32. Do you have difficulty concentrating at school?	118	0.544	0.40 - 0.66
33. Do you have difficulty concentrating at other times (e.g., playing cards, computer games or reading)	118	0.566	0.43 - 0.68
34. How often is homework or study hard for you?	116	0.638	0.52 - 0.73
35. How often do you need more help with school work than others in your class?	118	0.437	0.28 - 0.57
36. How much difficulty do you have remembering things at school/college or work?	117	0.550	0.41 - 0.66
37. How much difficulty do you have concentrating at work or school?	117	0.624	0.50 - 0.72
38. How much difficulty do you have with reading and writing?	117	0.582	0.45 - 0.69
39. How much difficulty do you have with math and calculation?	116	0.562	0.42 - 0.68
40. How much difficulty do you have with your school work, compared to others in your class?	116	0.591	0.46 - 0.70
41. I find it easy ti have an intimate relationship	116	0.612	0.48 - 0.71
42. I am confident when I am with people of the opposite sex	115	0.763	0.67 - 0.83
43. I am happy with the way things are	117	0.519	0.37 - 0.64
44. I am happy with life in general	116	0.589	0.46 - 0.70
45. In general, I am satisfied with my current life situation	117	0.516	0.37 - 0.64

## Discussion

This study assessed the psychometric properties of the Swedish version of the MMQL-Youth Form and Adolescent Form in a sample of Swedish children from 6^th^ and 9^th^ years in seven primary schools in a county in western Sweden. The results in the present study indicate that the Swedish versions of the MMQL-Youth Form and Adolescent Form are valid instrument for self-report measurement of Swedish children’s HRQoL. The questionnaires were validated in terms of face and content validity and participating children appraised that the items were relevant and that the questionnaires had sufficient clarity and readability. Children should be involved in instrument development and validation [10] and an explanation for why respondents felt the questionnaires easy to understand and to fill out could be that 20 children were involved in the translation and interpretation procedure. A strength in the present study is that the sample was large and diverse with regard to socioeconomic background by including children from schools in different neighborhoods and that the response rate was high. A reason for the high response rate could be that researchers stayed in the classroom during the data collection and informed the children about the objectives of the study. Informants gave positive responses on participation like, “it feels good to be able to help in such things”.

### MMQL-youth form

The internal consistency of the MMQL-Youth Form in this sample was found to be acceptable for the total scale with an alpha coefficient of 0.88. This Cronbach’s alpha value is quite similar to the first test of Cronbach’s alpha by Bhatia et al. [13]. An exception in the present study is the subscale Physical Functioning with an alpha coefficient of 0.66, not attaining the acceptable alpha of 0.70. In the first test by Bhatia et al. [13] this subscale reached an alpha coefficient of 0.78. A reason for this discrepancy could be that the respondents in the present study were healthy children and that some of the items are more valid when having an illness. A low internal consistency value on physical functioning has also been found in other studies with children [23] and future research will be important to investigate if internal consistency differs in samples of children affected by disease. The internal consistency of the MMQL-Youth Form in the present study is comparable with other instruments such as PedsQL™ and the European KIDSCREEN-52 for the measurement of HRQoL for children [11,24]. The stability of the scale over time with a two-week span that is deemed to be suitable for assessing the stability of a questionnaire [18] showed that 50% of the items in the MMQL-Youth Form demonstrated good agreement and 50% moderate agreement between the test and retest. The ICC values for the MMQL-Youth Form in the first test by Bhatia et al. [13] also showed moderate to good agreement between the test and retest (ICC ranged from 0.56- 0.79). The ICC values for the KIDSCREEN-52 ranged from 0.56-0.77 [11] which is quite similar to the ICC values in the test of MMQL-Youth Form by Bhatia et al. [13] and in the present study.

Both Bhatia et al. [13] and Shankar et al. [14] administered the questionnaire MMQL-Youth Form to the children (8 to 12 years old) in the form of face-to-face interviews. In the present study children were in the 6^th^ year of primary school and completed the MMQL-Youth Form by themselves. However, researchers and/or teachers were present in the classroom and when needed could explain the items for the children. Our procedure shows that it is feasible to let the children in this age group answering the questionnaire independently when they have the possibility to have the items explained. This is supported in a review by Riley [25] who found that children in this age group can successfully self-report age-appropriate health related quality of life questionnaires. Whether also younger children (8 to 10 years old) can complete the MMQL-Youth Form independently and what support is needed for this could be the focus of future studies. Interview-administered questionnaires can be applied when the children are unable to read or write and can have some advantages. The number of items omitted can be reduced and the interviewer has the possibility of rephrasing the question or to probing for a more complete response [18]. However, more time and cost efficient alternatives of administration, like the one described here, increase the usefulness of the instrument.

### MMQL-adolescent form

The internal consistency of the MMQL-Adolescent Form in this sample was found to be acceptable for the total scale as well as for the subscales. The alpha coefficient of 0.92 for the total scale is indicating a high level of reliability and is the same as for the first test by Bhatia et al. [12]. The internal consistency of the Adolescent Form in the present study can be compared with other instruments for measurement of HRQoL for similar age group such as the CHQ-CF instrument with an alpha coefficient between 0.69-0.92 for the subscales [26]. The stability of the scale over time with a two-week span showed that the ICC ranged between 0.248 and 0.763, with 80% of the items having moderate or good agreement and 20% fair agreement between the test and retest. In the test of the MMQL-Adolescent Form by Bhatia et al. [12] the ICC score ranged between 0.60 – 0.90 for the subscales. ICC values for each item are, to our knowledge, not available [12]. Having only a fair agreement level for some items may be due to a number of issues, for example, changing perceptions of informants between the first and second measurement. Conditions that may change over short intervals are, for example, anxiety, mood or pain [18,22]. This could thus indicate that the instrument is sensitive to changes and that a low ICC value does not necessarily mean that the instrument is unstable. This explanation is supported by the fact that feelings appear to change quickly among adolescents dependent on what happens in their everyday life [27]. There is thus a need for further research of stability of the Swedish version of MMQL-Adolescent Form.

Some methodological considerations should be discussed. The ICC values were only fair for some subscales in the MMQL-Adolescent Form, it can be seen as a strength that the instrument captures mild changes over time. However, the results would have been strengthened if the children taking part in the retest were asked to respond to whether they felt better, worse or the same. Then the test-retest reliability could have been evaluated based on the children who reported no change in wellbeing. Future studies comparing children reporting change or no change in wellbeing within a short time frame would be important to evaluate the responsiveness of the instrument to mild changes. Evaluating the discriminate validity was beyond the scope of this study. In another study the instruments were able to distinguish between HRQoL in children with different age, gender and socio-economic conditions both for MMQL-Youth Form as well as for MMQL-Adolescent Form (unpublished observation, Hutton et al., submitted April 2013). Additional studies including children with different diagnosis i.e. cancer will be required to establish if the instrument is reliable and valid for subjects with different conditions especially if combined with a construct validity evaluation.

## Conclusion

In conclusion, the present study of the psychometric properties of the Swedish versions of the MMQL-Youth Form and the Adolescent Form shows that the Swedish versions are valid and reliable in this age group (11–16 years old) and in the context of healthy school children. The original development of the both instruments is based on extensive exploratory work with the focus of investigating the long term effects on HRQoL of children with experience of cancer. Sound psychometric properties in this study support the use of the MMQL instrument in healthy populations and thus also in studies of children who return to a normal life upon cancer survival. It is recommended that our studies are extended to investigate the instrument among diverse samples of children to evaluate its usefulness in clinical settings to assess health care and to identify children in need of support. It would also be of interest to perform correlation studies to test if QoL correlates with other dimensions of health.

## Consent

Written informed consent was obtained from the patient for publication of this report and any accompanying images.

## Competing interest

The authors declare that they have no competing interest.

## Authors’ contribution

All of the authors have contributed in this study with design and instrument development, data collection and interpretation of the results as well as contributed in article writing to the final version. The study was originally designed by PS and JN. The manuscript was drafted by E-LE with supervision of PS and JN. Critical revisions for significant intellectual content were made by all authors. All authors read and approved the final manuscript.
